# Visual pH Sensors: From a Chemical Perspective to New Bioengineered Materials

**DOI:** 10.3390/molecules26102952

**Published:** 2021-05-16

**Authors:** Luigi Di Costanzo, Barbara Panunzi

**Affiliations:** Department of Agricultural Sciences, University of Naples Federico II, 100, 80055 Portici, Italy; Barbara.panunzi@unina.it

**Keywords:** pH, optical sensors, fluorescence, colorimetric, MOF, GFP

## Abstract

Many human activities and cellular functions depend upon precise pH values, and pH monitoring is considered a fundamental task. Colorimetric and fluorescence sensors for pH measurements are chemical and biochemical tools able to sense protons and produce a visible signal. These pH sensors are gaining widespread attention as non-destructive tools, visible to the human eye, that are capable of a real-time and in-situ response. Optical “visual” sensors are expanding researchers’ interests in many chemical contexts and are routinely used for biological, environmental, and medical applications. In this review we provide an overview of trending colorimetric, fluorescent, or dual-mode responsive visual pH sensors. These sensors include molecular synthetic organic sensors, metal organic frameworks (MOF), engineered sensing nanomaterials, and bioengineered sensors. We review different typological chemical entities of visual pH sensors, three-dimensional structures, and signaling mechanisms for pH sensing and applications; developed in the past five years. The progression of this review from simple organic molecules to biological macromolecules seeks to benefit beginners and scientists embarking on a project of pH sensing development, who needs background information and a quick update on advances in the field. Lessons learned from these tools will aid pH determination projects and provide new ways of thinking for cell bioimaging or other cutting-edge in vivo applications.

## 1. Introduction

Optical chemical sensors are considered “eyes capable of seeing beyond the human perception” in that offer advantages to human sight and can be regarded as a miniaturized real-time analytical devices [1]. Optical sensors represent great analytical tools for technologies requiring real time continuous monitoring and gained researchers’ interest in many chemical contexts. They are routinely used for biological, environmental, and medicinal applications [1,2]. 

**Scheme 1:**
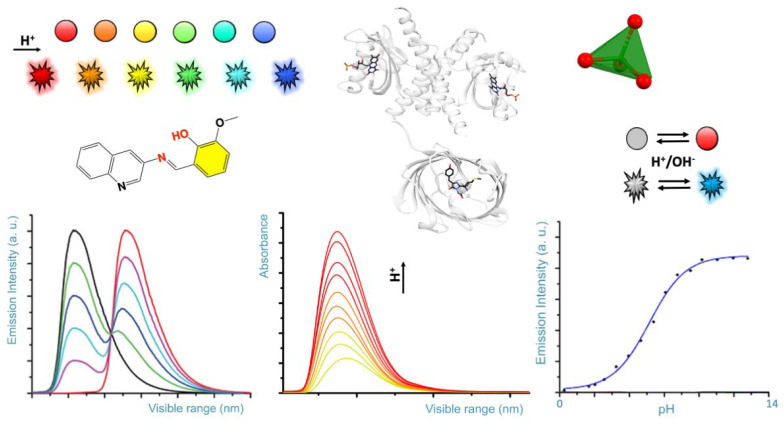
Rendition of typical colorimetric variation of pH sensors (colored spheres), and fluorescence emission as function of the pH (colored flashing shapes). Typical, spectral diagrams observed for the emission and absorbance as function of the pH and for a given wavelength are represented on the bottom.

Optical transduction techniques are consequence of the selective change of their optical properties on interaction with the analyte. Absorption and fluorescence emission spectral patterns are among the most explored optical properties. Spectral absorbance is defined as the logarithm of the ratio between transmitted spectral radiant power through a material and the incident radiation. The absorbance spectroscopy is an analytical technique based on the measurement of the absorption of radiation across the electromagnetic spectrum as function of wavelength of radiating light. The result is called absorption spectrum of the sample. On the other hand, when irradiated with an exciting radiation, a chemical compound can produce its own emission spectrum. Emission is the electromagnetic radiation produced by the electrons of the molecule when transitioning from a higher energy state to a lower one. There are several possible electron transitions for each atom/molecule, with specific energy difference that represent the emission spectrum. Fluorescence emission occurs to relax an excited singlet state of an atom/molecule, upon absorption of a radiation, to a ground singlet state and the overall spin is conserved. Upon returning to the ground state, the excited atom/molecule emits a photon of lower energy and longer wavelength than the absorbed photon. The radiation used to promote the emission is called “excitation” wavelength, while the resulting radiation is known as “fluorescence”. Typically, the excitation wavelength is represented by the absorbance peak wavelength. The difference between the “excitation” and the “fluorescence” peak is known as “Stokes-shift”. Large Stokes-shifts improve fluorescence signal due to a decreased overlap between the absorbance and the emission bands [3]. Fluorescence and absorption spectroscopy can be considered complementary techniques and provide response in the visible spectral region and are employed as analytical chemistry tools to quantify the amount of a particular analyte in the sample [4].The first stage of an optical sensor transduction involves a chemical interaction between the analyte and a transductor to produce an optically detectable signal. Substances able to produce absorbance/fluorescence spectra in the visible region up to near infrared (NIR) region are typically named chromophores/fluorophores [5,6]. These substances are organic or hybrid molecules with an electronic configuration capable of transitions in the visible spectrum. For our aims, we focus on sensors producing optical signals within the visible spectrum and detectable in absorbance and/or in fluorescence. Specifically, we refer to colorimetric sensors as a class of optical sensors able to change their color when stimulated with natural light, and fluorescence sensors able to change intensity and/or emission color when stimulated with UV-light [7]. Among colorimetric sensors, we refer to “visual sensors” (abbreviated as VSs) since, similar to an “optical instrument”, the eyes are able to detect the signal produced by these molecules [8,9,10,11]. These sensors offer an advantage over spectrophotometric and electroanalytical analysis, more expensive and time consuming [12,13]. VSs can offer real-time and *in-situ* sensing, even in remote sensing mode, and non-destructive analysis. Typically, VSs are classified into *on-off* and gradual sensors, respectively [14,15,16,17]. For *on-off* VSs, two different colors or different emissions are detected between low and high pH values [17]. For gradual VSs a progressive colorimetric/fluorimetric response is obtained corresponding to an increase of the analyte concertation (Scheme 1) [18,19]. If the sensor emission can be stimulated by two excitation wavelengths, it will be referred as “ratiometric” sensor [20].

Hints from history help us to understand how visual pH sensors research is evolving. In 1907 Henderson published a paper describing the relation between the concentration of hydrogen ion [H^+^] and buffer composition. In 1909, Soren Peder Lauritz Sørenson proposed the more convenient pH and pK_a_ definitions as the negative logarithm of [H^+^] and of the equilibrium constant, respectively. Although Henderson defined the equilibrium constant, Ka, in terms of a concentration ratio in 1908, it was in 1916 when Hasselbalch proposed a recognized relationship between pH and pK_a_ known as the Henderson-Hasselbalch equation. This equation relates the two parameters to the equilibrium concentrations of a dissociated acid [A^−^] and its non-dissociated acid form [HA], respectively:

pH = pKa + log ([A^−^]/[HA]) (1)

This equation allows to deduce pH sensor ability of a molecule knowing its Ka value. Sørenson pairing of a hydrogen electrode with a calomel reference electrode was followed by the 1920 Duncan McInnes and Malcolm Dole first glass electrode, equipped with a glass semipermeable membrane. The potential of a hydrogen electrode (proportional to “H^+^ activity”) dipped into the analyte solution represents the signal that is correlated to the measured pH value. 

Since the first pH measurement in 1909 by Sørenson, development of more selective sensors of proton H_3_O^+^ (simply H^+^) concentration in solution still fascinates chemists [21]. From the earliest pH sensors to modern pH chemosensors over a century has passed. Today, we recognise pH as a fundamental parameter of both chemical and biological interest. Enzymatic reactions as well as overall cellular buffer systems function within a narrow and specific pH range. Deviation from this value can help to diagnosis as for the acidic environment of cancer cells [22]. Therefore, monitoring of pH within biological systems represents a great goal for the ongoing “pH sensors” research and it is not surprising the growing researchers’ interests for design, synthesis, chemical characterization, and sensing mode. A preliminary stage for sensor development involves custom made sensors, not necessarily targeted for biological purposes. These sensors can be used as starting point for further modification of functionally sensing needed for biological and biomedical use. Today, the scientific frontier is represented by bio-engineered, nanosized, and multi-channel systems capable of non-invasive on-site analysis. 

Despite several studies encompassing the topic of visual pH sensors, we report an overview of the most recent types of developed visual pH sensors considering different typological and chemical entities and three-dimensional structure. In this review, we focus on the state-of-the-art of visual pH sensors (abbreviated as pH VSs) developed over the past five years (years 2016–2021) based on synthetic novelty, study completeness, optical selective response, theoretical deepening, and biochemical applications. Specifically, we review the literature and organize the discussion around pH VSs in the following sections (also represented in Scheme 1):

(1) Molecular synthetic organic sensors;
(2) Metal organic framework (MOF)-based sensors;
(3) Sensors from engineered nanomaterials;
(4) Bioengineered sensors.

Primary literature and reviews were consulted to understand the structure, sensing configuration, operating mode, and optical response. Specifically, we discuss a selection of most relevant and innovative *on-off*, gradual, and/or ratiometric visual pH sensors (colorimetric, fluorescent, or dual-mode responsive). We further underline the representative applications of visual pH sensors targeted for biological and medical scope. 

## 2. Mechanisms of Fluorescence and Colorimetric pH Sensing

Several mechanisms explain the fluorescence, including photoinduced electron transfer (PET), internal charge transfer (ICT), Förster (or fluorescence) resonance energy transfer (FRET), excited state intramolecular proton transfer (ESIPT) or other formation of excited states able to produce photon emission [21,23,24,25].

Photoinduced electron transfer (PET) is an excited state electron transfer process occurring in the same molecular system and as consequence of an electron transfer from electron-donor to an acceptor-donor atom via non-radiative pathway. PET process can quench fluorescence signal of an organic molecule. In many sensors, PET process channels signal to the analyte that in turn results in an emission of fluorescence [26].

Intramolecular charge transfer (ICT) is a mechanism occurring for fluorescent sensors when an electron-donating group of the fluorophore sensor is linked to an electron-acceptor group. During sensing processes, the sensor electron density is greatly affected by the analyte resulting in significant shifts in the absorption and fluorescence emission bands. Molecules with enhanced acidity or basicity in the excited state undergoing an intramolecular excited-state proton transfer (ESIPT) process in presence of the analyte are ideal candidates as pH sensors.

Energy transfer by resonance (FRET, Fluorescence or Förster Resonance Energy Transfer) represents an energy transfer between fluorophores. FRET is useful to obtain structural information of biological molecules and this spectroscopic technique allows to identify the distance between two molecules with extreme precision. FRET mechanism explores the mutual interference of fluorescent molecules, called donor and acceptor, bound to the biological molecule of interest [27]. The donor molecule such as a pH sensor can be excited with a specific wavelength and its emission energy in turn can be transmitted to the acceptor molecule. FRET energy transfer can occur if the molecules are in close proximity. Finally, if the acceptor molecule gives an emission, it can be measured by the operator.

## 3. Synthetic Visual pH Sensors

Early traditional pH sensor molecules were extracted from flowers, fruits, and vegetables as sustainable and non-toxic natural dyes (colored molecules). For example, an efficient colorimetric pH sensor based on anthocyanin was recently extracted from purple sweet potato [28,29]. These established sensors were followed by novel synthetic chromophores/fluorophores functioning as pH sensitive compounds. These molecules meet specific analytical requirements such as photostability, water solubility, large lifetime, Stokes shifts, and fluorescence quantum yields. The newest synthetic sensors are often guided by logic of low-cost process, environmental sustainability, and biocompatibility.

The first step for the development of novel pH VSs, is represented by synthesis of simple small organic molecules or organic polymeric systems. These pH VSs can function in an *on-off*, or in gradual and/or ratiometric mode. For analytical purposes, especially in aqueous solution, *on-off* pH sensors provide a rapid and sharp change of color around a precise pH value that allows for pH monitoring. On the opposite, for many biological samples, where a gradual and small change around physiological pH (or a different value) is experienced, gradual and/or ratiometric pH VSs are more suitable [30,31,32]. Ratiometric sensors allow for measurements of two emission wavelengths in order to calculate the ratio of fluorescent intensities for a high measurement accuracy [33]. This ratio has the advantage of being independent by the total fluorescence intensity, making the signal correction for optical interference (readout system, photobleaching, inhomogeneity) no longer necessary and therefore, of easier and immediate application. A discussion of molecular organic pH VSs will follow in Section 3.1.

### 3.1. Molecular Organic Visual pH Sensors

**Figure 1:**
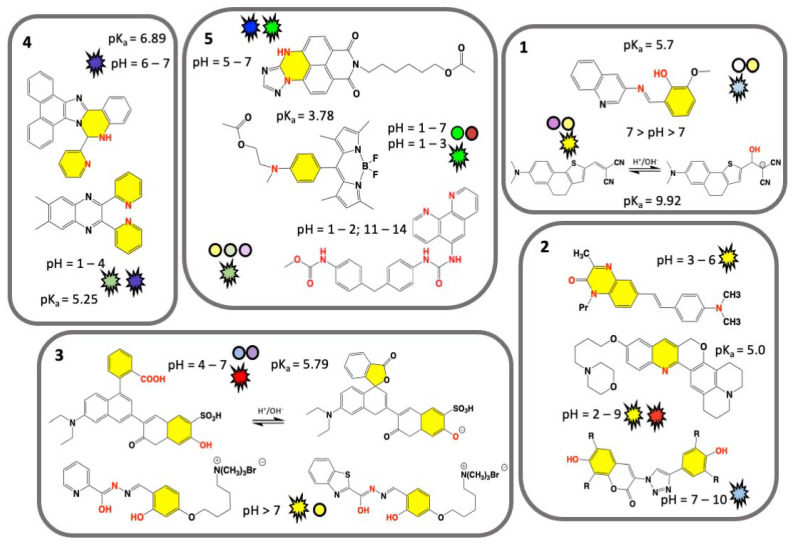
Chemical structures of recent synthetic molecular organic visual pH sensors grouped on the base of a common visual response or chemical features. Nitrogen and oxygen atom groups responsible for protonation/deprotonation are marked in red, and their chemical ring(s) marked in yellow. Sensor colorimetric response and pH value are represented by a colored sphere, while fluorescence emission by a flashing shape. Color matches to the sensor real color within the displayed pH range. Unique value of pKa is displayed. Box 1. Classical colorimetric and fluorescent pH VSs [22,40]. Box 2. Predominant fluorescent pH VSs [41,42,43]. Box 3. Designed water-soluble pH VSs [44,45,46]. Box 4. AIEgens pH VSs [47,48]. Box 5. Polymeric pH VSs [49,50,51,52,53]. Adapted from references indicated in square brackets.

Electrochemical pH sensors development was achieved with significant results in last few decades for the measurement of potential, current, or impedance of a selective electrode. These are primarily made of inorganic materials (e.g., glasses), or in combination with nanomaterials (e.g., doped membranes) [34,35]. On the opposite, low and high molecular weight organic chemosensors are designed for measurements of pH value and utilize easily exchangeable protonation/deprotonation equilibria (or metal ions) of nitrogen and oxygen atom groups [36]. These sensors can also measure the pH of biological samples including the pH value of subcellular locations on different scale [37]. Tunable chemical fingerprint and sensing mechanisms can be achieved by chemical branching modification of a pH sensitive dye core’s molecule fragment. Polymeric pH VSs, obtained by linking of several sensing organic molecules, used as monomers, can be fixed to a material of support and are ready for potential functional applications. In this section, we discuss the recent well characterised molecular organic VSs functioning in colorimetric and/or fluorescence mode, by *on-off* or gradual and/or ratiometric response [38,39]. We examine their chemical mechanisms and provide considerations for their potential use. A selection of synthetic pH VSs is summarised in Figure 1 (boxes 1–5) and is grouped based on common visual response and/or chemical features. For a quick glance, nitrogen and oxygen atom groups responsible for accepting or donating hydrogen atom for protonation/deprotonation are marked in red, and their linked chemical ring(s) marked in yellow. Sensor colorimetric response is represented by colored spheres (see Figure 1) and corresponding fluorescence emission colors (as visually experienced) is represented by flashing shapes. 

Two classical pH VSs are illustrated in Figure 1 (box 1) producing absorbance and fluorescence colorimetric response from acidic to basic medium. A pH VS was chemically synthesised by condensation of 3-aminoquinoline Schiff base with *o*-vanillin and fully characterized by A. Saha and coworkers [22]. Obtained as an “easy-to-make” organic sensor, it functions as an acidic pH fluorescence and colorimetric sensor. Colorimetrically the sensor exhibits two bands at 330 nm and 400 nm at pH 7.0, associated to n-p* and p-p* transitions, respectively. The intensity of the absorption band at 330 nm gradually increases with decreasing of pH from 7.0 to 2.0, while a concomitant decrease of the absorption band at 400 nm is measured. As for the fluorescence response, the sensor exhibits naked-eye perceivable color change from acidic to basic pH under a UV-lamp (Figure 1, box 1). The fluorescence is quenched by PET mechanism resulting from protonation of the imine nitrogen group. Deprotonation of the same nitrogen cause locking of PET mechanism which in turn results in restoration of fluorescence with a neat *on-off* performance [22]. This sensor gives-off a fluorescence signal change between normal and cancer cells, as function of pH in vitro cytotoxicity [22]. A similar colorimetric and fluorescence turn-on pH sensor is comprised of a highly colorimetric naphthalenone scaffold (Figure 1, box 1) and studied by S. Wu and coworkers [40]. This sensor shows a color change from light purple to yellow (acid to basic), and a fluorescence signal in the pH range of 9.0–14.0, with an emission from colorless to orange, and finally to yellow (box 1) [40]. This response is associated to an ICT mechanism induced by structural changes caused by -OH group binding, which translate a non-fluorescent molecule into a highly emissive form. Fluorescence imaging of HeLa cell line reveals a good cell membrane permeability, and its ability for selective monitoring even of extreme basic pH variation [40].

Predominant fluorescent pH VSs are illustrated in Figure 1 (box 2). A series of six quinoxalin-2-ones dyes with *N,N*-dialkylaminostyryl substituents in different combinations were studied by T.P. Gerasimova and coworkers [41]. For pH-sensing in aqueous solution the dyes were encapsulated into L-α-phosphatidylcholine based bilayers. pH sensitive emission reveals significant effect of the dyes structure/interaction in the bilayer and produces a maximum sensing response within pH range of 3.0–6.0. Quantum chemical calculation revealed that the protonation mechanism undergoes different paths depending on the alkyl group of the *N,N*-dialkylaminostyryl substituent and/or the position of a donor substituent relative to the quinoxalin-2-one [41]. A lysosome-targeting sensor (named CQ-Lyso) based on the chromenoquinoline chromorphore gave ratiometric fluorescence response to intracellular acidic pH in living cells (box 2) [42]. This sensor was developed as a pH VS: in acidic media, the protonation of the quinoline ring induces an enhanced intramolecular charge transfer (ICT). This process results in a yellow fluorescence in neutral/basic media (pH 7.4), and a change from yellow to red/brown color using the same excitation wavelength at pH 4.0 [42]. 

A limited number of pH sensors with an *off-on-off* fluorescence signal variation have been reported to date, potentially more useful than conventional *on-off* sensors. Sensors with *off-on-off* behavior can detect a selected pH range within the whole pH range in which their signals are visible (-*on* state). Among *off-on-off* fluorescence sensors, the one consisting of a coumarin group fused to a triazole ring was developed (box 2) by T. Hirano and coworkers [43]. This sensor can be used for fluorescence imaging of pH within intracellular human mcf-7 cell line (cancer cell) and it turns fluorescent at pH 6.0, but not at pH 8.0; suggesting a use in microenvironment living cells [43]. 

Specifically designed water-soluble pH VSs are illustrated in Figure 1 (box 3). Water solubility is a key requirement for biological applications and water-soluble visual pH sensors are uncommon and highly required. To obtain a water-soluble sensor specific ionic groups such as sulphonic or quaternary ammonium groups are attached to the sensing molecule. A water-soluble pH sensor for sensing and imaging was obtained by introducing a sulfonic group to the chemical structure of condensate rings by A. Zheng and coworkers [44]. The synthesized sensor contains a pH-responsive phenol group undergoing deprotonation (box 3), that in turn improves the ICT mechanism [44]. In basic condition an intramolecular spiro-cyclization and decreased of π-conjugation of the sensor occur. As a result, the maximum absorption of the system is red-shifted, and the fluorescence is enhanced. The sensor can be used for pH sensing through its absorption or fluorescent signals, and a linear relationship was detected. Fluorescence imaging and cell viability of human cancer HepG2 cell line in buffer with pH between 4.5 and 7.4 show a clear fluorescence enhancement [44]. 

Sensing ability based on small water-soluble, simple, and organic molecules attracts researchers’ interest [45,46]. By adding on the sensor’s core a flexible five methylene groups branch bearing a charged trimethylammonium bromide group (box 3) B. Panunzi and coworkers developed two water soluble sensors, while preserving their organic phase solubility. These sensors are based on a substituted aroyl-hydrazide skeleton undergoing to tautomeric equilibria depending on pH value. In these molecules colorimetric effect is the result of ESIPT mechanism. Owing their amphiphilic nature, the charged sensors could potentially interact with cell membrane without altering the bilayer organization [40]. These sensors show naked-eye *on-off* switch at neutral pH, absorption and fluorescence response, high sensitivity. 

Beginning with the pioneering studies of Tang and Park in 2001 a unique class of molecules called AIEgens (aggregation-induced emission) was developed [54,55]. Typically, AIEgens molecules contain multi-aromatic conjugated rings with potentially free rotating chemical groups. Although weakly or not emissive as isolated molecules, they become emissive in the solid state, or in concentrated solution, due to intermolecular processes and as consequence of their restriction of intramolecular rotation (RIR) [55]. AIEgens molecules have shown properties as fluorophores and exhibit aggregation-induced emission, or emission enhancement. The aggregate state of the AIE molecules, often characterized by X-ray structural studies, is the result of hydrogen-bonding, p-stacking, or other Van der Waals interactions [55]. These unique characteristics differentiate AIEgens from conventional luminophores and are currently explored as optical sensors including specific interactions derived by samples pH changes. Recent examples of AIEgens pH VSs are illustrated in Figure 1 (box 4). A visual AIEgen pH comprised of a (2-pyridyl)-quinoxaline dye with two free twisting pyridyl rings in 2 and 3 positions of the quinoxaline moiety sensor was developed by A. Misra and coworkers (Figure 1, box 4) [47]. The molecule exhibits an AIE enhancement triggered by proton addition. As a result, the sensor solution change from colorless to blue color in the aggregate state and under UV lamp. Furthermore, protonation causes a reversible fluorescence switch between basic and acidic conditions and the emission color changes from blue to green in the aggregate hydrosol state (*aka* a colloidal mixture having water as the dispersion medium). The blue shift is observed in both absorption and emission spectra and theoretical calculations served to deduce the mechanism. A more recent AIEgen visual pH sensor is represented by the phenanthro-imidazole-based sensor for dual-responsive turn-on detection of acidic pH by Q. Deng and coworkers (box 4) [48]. This sensor is also able to recognize copper ions. The weakly fluorescence sensor exhibits typical AIE turn-on phenomenon in water/ethanol mixture. The sensor was successfully employed in an aggregate form for acidic pH turn-on in HeLa cells, proving to be useful for monitoring cellular pH.

### 3.2. Polymeric Organic Visual pH Sensors

Trending methods of pH detection involve polymeric materials, widely explored for biomedical and environmental applications [56]. Comprehensive review papers are available and discuss organic micro- and macro-scaled organic pH sensors including healthcare applications [57]. In most cases pH sensing involves electric and electrochemical methods [57,58]. However, there are only few polymeric pH sensors exploring absorption and fluorescence response. Many polymeric pH VSs are obtained by immobilization/entrapping of dyes on materials including silicate, polyvinylidene fluoride (PVDF), cellulose acetate [59]. One approach is represented by the use of a fluorescent polymeric micelle, consisting of a pH sensitive fluorophore linked to a fatty acid molecule, and able to self-assemble in a vesicle-like envelope inside the cell, able to sense a pH variation [60]. On the other hand, polymeric pH VSs can be obtained by covalent linkage of fluorophores/chromophores through several strategies of polymerization or copolymerization [61,62,63]. A representative polymeric pH VS was developed by Y. Tian and coworkers in 2013 by copolymerization of monomeric units of fluorescein (green color) and/or dihydrofuran (red color) in methacrylate and acrylamide copolymers and used as a biocompatible material for drug delivery, bioimaging, and biosensing [64]. Recently developed polymeric pH VSs are illustrated in Figure 1 (box 5).

A classic pH sensor containing five monomeric units of triazole heterocycle fused in a five-condensed rings unit, and conjugated to a methacrylate and acrylamide copolymer, was developed by Y. Tian and coworkers [49]. This mixed sensor/copolymer functions as a weak electron-donor group in acidic conditions, while in basic conditions, the -NH group is deprotonated resulting in stronger electron-donating ability of the triazole unit. The polymer optical property is the result of an ICT mechanism triggered by protonation [49]. The pH sensor copolymer shows a strong fluorescence-based ratiometric pH response, exhibiting blue emission around 477 nm in acidic conditions, and green emission around 507 nm in basic conditions. Fluorescence responsive fragments were prepared as monomers which were polymerized to form a fluorescence responsive polymer. The polymer has the potential for monitoring cellular pH value as well as pH distribution in imaging techniques. 

A different approach was used by H. Lee and coworkers to develop a polymeric pH fluorometric chemosensor containing a monomeric unit of difluoroboron dipyrromethene (Figure 1, box 5) [50,51]. This sensor exhibits excellent solubility and stability in water, and in acidic conditions shows an intensity enhancement of 4.6 times with respect to the unprotonated sensor. This characteristic is consequence of PET suppression mechanism due to electron-donating of the sensor’s tertiary amine to its difluoroboron dipyrromethene skeleton group [51]. The response in acidic pH, was measured by fluorescence imaging for in vitro cells and *E. coli* bacteria (surviving in acidic pH) and paper-strip embedded with the polymer was used to test practical *in-situ* applications. 

A highly sensitive and pH-responsive polymeric system (named Phen-MDI-CA) represented by a chemical engineered sensor on a natural substrate was developed by J. Zhang and coworkers [52,53]. Specifically, a cellulose was used as a skeleton, urea group as an anchoring bridge, and phenanthroline group either as a chromophore or a metal ion coordinating group. The Fe(II) cations can act as cross-linking agents among cellulose polymeric chains. Therefore, Phen-MDI-CA functions as sensor for iron cations while being responsive in a wide pH range [52]. Colorimetrically, it discriminates within pH range 11.0–14.0 on paper stripes test, and pH 1.0–2.0 by fluorescence [53]. Three ratiometric systems were obtained by mixing Phen-MDI-CA with pH-responsive dyes used as reference, consisting of *meso*-tetraphenylporphyrin, a protoporphyrin derivative, and malachite green in DMSO. Specifically, these ratiometric mixtures display different emission colors within a small range of 0.2–0.4 pH units within the overall pH ranges of 11.0–14.0 and 1.0–2.0. Therefore, these ratiometric systems amplify color difference allowing for a good measurement accuracy.

### 3.3. Nucleic Acids Visual pH Sensors

An interesting class of polymeric pH VSs is represented by nucleic acids and can be targeted for pH sensing and combined to dye molecules to form pH sensors. One approach is based on the use of functionalized short singled-stranded nucleic acid sequences (30–50 bases) of RNA or DNA able to form hairpin structures upon annealing with a complementary oligonucleotide chain. By covalently linking a pyrene group to both 5′ and 3′ ends of an oligonucleotide B. Juskowiak and coworkers were able to form a so-called molecular beacon (MB). This dual-labelled oligonucleotide was used as a pH fluorescent sensor because emitting in acidic pH (emission ~ 480 nm) [65]. When pH decreases, protonation of cytosine nucleotide causes a switch to a folded tetraplex structure. As consequence, MB sensor resulted in an efficient reversible DNA-based switch for real-time intracellular pH monitoring of live HeLa cells.

## 4. Nano-Sized Visual pH Sensors

Nanomaterials are materials with characteristic geometric patterns inspired by fullerene (a carbon allotrope) and one dimension on the scale of nanometer (one billion nanometers (10^9^) in one meter) and in the typical range of ~1–~100 nanometers. Since characterization of graphene (another carbon allotrope), nanomaterials showed surprising magnetic, electrical, and optical properties [66,67,68]. Nanomaterials based on graphene are used to engineer novel generations of chemo- and bio-sensors for recognition of phenomena occurring at nanoscale level [69]. These materials have great impact in biological and biochemical applications such as pH measurement in cells, where micro and nanoscale measurement within cellular compartments are needed [68,69,70].

Dyes made of chemical fragments optically responsive to pH can be linked to nanoparticles for targeted delivery. Nanoscaled pH sensors are widely studied for bioimaging and optical sensing including nanosized metal-organic frameworks (MOFs), carbon dots and graphene quantum dots (GQDs), inspired by the fluorescent properties of carbon nanoparticles derived from candle soot in presence of nitric acid [71,72,73,74,75,76,77].

**Figure 2:**
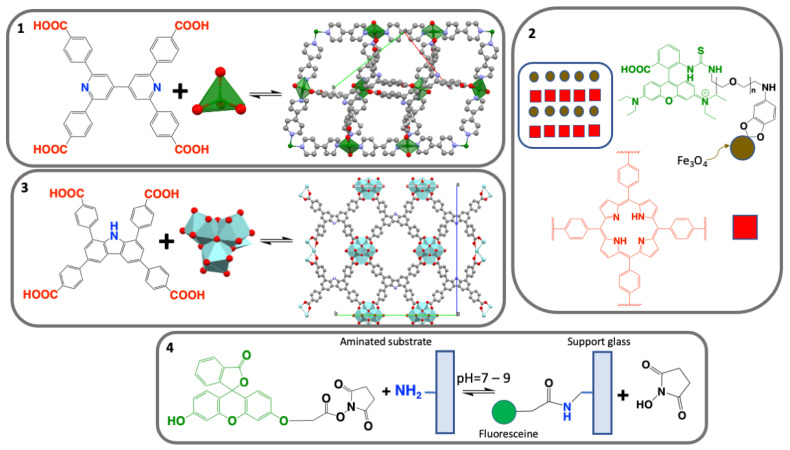
Recent nano-sized visual pH sensors. Box 1. MOF obtained from bipyridyl-tetracarboxylic in complex with Zn(II)-oxo specie derivative (green tetrahedral pyramid, oxygen red spheres, nitrogen blue spheres). Three-dimensional structure is obtained from Cambridge Structural Database (CSD) [83] with accession code 2019034 [84] Box 2. A nanocomposite pH VS is obtained from rhodamine B bound to a 3,4-dihydroxybenzaldehyde-polyethene glycol amino (DBI-PEG-NH_2_) functionalized with Fe_3_O_4_ (colored brown sphere) [85]. This composite is then mixed to *meso*-tetra (4-carboxyphenyl) porphyrin (red squares), and the overall mixture dispersed into a zirconium-based MOF (highlighted box) [86]. Box 3. MOF named BUT-62 is obtained from a fluorescent asymmetric ligand mixed to a zirconium oxo-complex (Zr cyan-polyhedron, O red spheres, N blue spheres; accession code 1446514) Box 4. pH VS from highly engineered nanomaterials. A “grafting” reaction causes binding of fluorescein molecule, as succinimidyl ester, to a glass (semi-transparent blue rectangle) coated with (3-aminopropyl)trimethoxysilane (APTMS, blue segment) [87]. Adapted from references indicated in square brackets.

MOFs are organometallic lattices consisting of single metal ions or metal clusters coordinated to one or more rigid organic ligands leading to linear, bidimensional or three-dimensional structures with high nano-porosity [78,79,80]. An infinite number of highly engineered structural combinations can be formed starting from a variety of organic and inorganic components. MOFs are excellent candidates for sensing purposes aiming the empty space contoured by their cavities. These spaces form pores that are potentially selective for analytes. Luminescent metal-organic frameworks (LMOFs) with luminescent fragments encapsulated in the lattice framework were successfully applied for analytical purposes and specifically for pH measurements [81]. Examples of MOFs were applied to intracellular pH ratiometric measurement. M. Shamsipur and coworkers provide a review of the current strategies used to develop nanosized systems (up to 2019) for fluorescence pH sensing [82]. Following with this review, we provide a chemical overview of the most significant recent examples of nanosized and MOFs pH VSs. Representative nano-sized pH VSs discussed below are illustrated in Figure 2. Often an acronym is used to identify published or patented MOFs.

### 4.1. MOF and COF Based pH VSs

Forefront research of visual pH VSs is aiming to develop multi-channel MOF and COF nanosensors based on three desirable targets:

(1) MOFs designed to sense pH variation associated to another biological parameter (such as oxygen cellular level, or a particular metabolite); 

(2) MOFs able to give fluorescence with multiple wavelengths emission for a more accurate pH determination. 

(3) COFs are bi- or three-dimensional frameworks obtained by organic building blocks.

A MOF able to detect pH and a small metabolite as 3-nitropropionic acid (3-NPA), a highly toxic mycotoxin derived from moulding of sugar cane, was developed by E.-Q. Gao and coworkers (Figure 2, box 1) [84]. The three-dimensional structure of this MOF synthesized from a bipyridyl-tetracarboxylic in complex with Zn (II) ions reveals an interpenetrated diamond shaped framework (Figure 2, box 1). The -N atom of pyridyl groups facing the MOF cavities are available for protonation and are responsible for reversible *on-off* fluorescence response in aqueous solution in the pH range 5.4—6.2. The signal is associated to an activation of ICT transitions upon pyridyl protonation and to the locking of n → π* transitions of the aromatic pyridyl group. 

An elaborate MOF named Eu_0.034_ Tb_0.966_-NMOF and comprised of Eu^3+^, Tb^3+^ ions as pivotal metal cluster, fumaric acid, and oxalic acid as organic counterpart, was developed by G. Qian and coworkers [88]. This MOF exhibits significant pH-dependent luminescence emission based on Tb^3+^ (emission at 545 nm, ^5^D_4_ → ^7^F_5_ transition) and Eu^3+^ (emission at 618 nm, ^5^D_0_ → ^7^F_2_ transition). This MOF is also called “self-referenced” because it undergoes emission from the components of same MOF. These two emissions allow for an accurate pH measurement in the range of pH 3.0–7.0. Interestingly, fluorescent microscopy images of neuroendocrine PC12 cell line fixed, for 24 h, with Eu_0.034_ Tb_0.966_-NMOF shows microtubular cytoskeleton and nuclei as fluorescently stained by the sensor. 

A multi-channel ratiometric luminescent metal-organic frameworks (LMOF) was developed J. Gu and coworkers. This nanosensor is obtained by a one-pot reaction of pH sensing chemical entities and integration into a matrix [89]. Specifically, a mixture of fluorescein isothiocyanate, meso-tetra(4-carboxyphenyl)porphyrin and a dye (1,3,6,8-tetra(4-carboxylphenyl)pyrene) is simultaneously integrated into a MOF comprised of a Zr_6_O_4_(OH)_4_ matrix (known as UiO-66) and 1,4-benzodicarboxylic acid) nanoparticles [89]. Through a single excitation wavelength, potential blue, green (due to FRET effect), and red emission are available for ratiometric simultaneous intracellular pH and oxygen determination. The sensor was used for HeLa cells fluorescence imaging. Another red-green-blue (RGB) multi-channel for simultaneous detection of pH and oxygen is represented by a MOF developed by X. Lu and coworkers (Figure 2, box 2) [85]. This mixed fluorescent MOF is obtained by encapsulating nanoparticles of 3,4-dihydroxybenzaldehyde-polyethene glycol amino (DBI-PEG-NH_2_) functionalized with -Fe_3_O_4_ into a Zr-MOFs, and further reaction with rhodamine B isothiocyanates (RBITC commonly used as an acidic pH sensor). This composite is then mixed to a *meso*-tetra (4-carboxyphenyl) porphyrin, and the overall mixture dispersed into a Zr-based MOF (Figure 2, box 2). The pH VS named RB-PCN, exhibits two emissions using the same excitation wavelength: in the acidic condition at 575 nm due to rhodamine B, and in basic condition at 641 nm due to PCN-224 (porphyrin-derivative). Finally, the sensor exhibits a fluorescence response to a wide range of pH (1.0–11.0). Fluorescence imaging in HeLa cells reveals sensor’s ability to detect intracellular pH changes [85]. Finally, two MOFs giving-off fluorescence emission with different wavelengths, as function of a broad pH range (1.0–8.0), were developed by J.-R. Li and coworkers [86]. These sensors were obtained by a flexible Zr-MOF. The MOFs named Zr_6_O_4_(OH)_8_(H2O)_4_(CTTB)_2_ (BUT-62, Figure 2, box 3) and Zr_6_O_4_(OH)_8_(H_2_O)_4_(CTNA)_2_ (BUT-63) were synthesized from fluorescent asymmetrical ligands, 4,4′,4′’,4′’’-(9*H*-carbazole-1,3,6,8-tetrayl)-tetrabenzoic acid (H_4_CTTB) and 6,6′,6′’,6′’’-(9h-carbazole-1,3,6,8-tetrayl)tetrakis(2-naphthoic acid) (H_4_CTNA) [86]. Like MOF, covalent organic frameworks (COFs) are a class of structured porous polymers obtained by organic building blocks able to form bi- or three-dimensional crystalline structures forming storage and sensing space. S. Wang and coworkers developed a COF based on 8-hydroxyquinoline (COF-HQ) for reversible dual-mode pH sensing [90]. The COF was obtained by Schiff reaction of 2,5-bis[2-(quinolin-8-yloxy)ethoxy)terephthalaldehyde and 1,3,5-tri-(4-aminophenyl)benzene [90]. The synthesised COF-HQ shows a linear decreasing fluorescence intensity in the range of pH 1.0–5.0 as pH decreases. In absorbance mode, the same COF-HQ sensor shows a color changes from yellow to black with pH decreasing, therefore functioning as a dual mode sensor [90].

### 4.2. pH VSs from Highly Engineered Nanomaterials

Emerging intracellular pH fluorescent sensor for bioimaging and optical sensing are based on highly engineered nanomaterials represented by functionalized surface able to bind (or “graft”) a pH sensing ligand bearing a fluorescent chemical group. Depending on the surface material, and sensing ligands, different synthetic approach can be used. Glass supports or metal coated surfaces are generally employed to create a sensitive grafted organic layer of functionalized ligands with the advantage of the manipulability and processability of a macro-supported nanostructure. By using this approach, B. Wacogne and coworkers, developed a molecular porous nanolayer formed by amino-silanization on glass substrates using different functionalization strategies (Figure 2, box 4) [87]. A glass coated with (3-aminopropyl)trimethoxysilane (APTMS) and (3-aminopropyl)dimethoxymethylsilane (APDMS) was used as support for dye grafting (Figure 2, box 4), a chemical reaction used to covalent link a sensing molecule to the supported coat. Specifically, a pH sensitive fluorescein molecule, linked to a succinimidyl ester, is covalently linked to the free amine groups of the silane layer through a “grafting”. As a result, a pH sensing amidic branching group is formed (Figure 2, box 4). The pH sensing experiments are based on pH dependent fluorescence spectra recorded with an optical fiber reflection sensor.

Intracellular pH fluorescent sensors such as quantum dots (QDs) derived from two dimensional materials as carbon dots and graphene quantum dots have recently emerged as fluorescence sensors for bioimaging and optical sensing. H. Song and coworkers fabricated a new class of Ti_3_C_2_ QDs coated with polyethylenimine [80]. These QDs exhibit a pH-dependent blue photoluminescence, due to the combination of the bandgap transitions and the surface defect emissions (leading to a fluorescence quantum yield of 7.13%).

The pH dependent emission was attributed to the absorption decrease/increase of the non-radiation rate induced by the deprotonation of its surface defects. The sensor was employed as a ratiometric VS to monitor the intracellular pH by confocal laser scanning microscopy technique.

Carbon dots (CDs) based materials show great potential for bioimaging studies of intracellular pH sensing due to their stable optical properties, biocompatibility, and low toxicity. A pH-sensitive CD for intracellular pH sensing by fluorescence lifetime imaging microscopy was developed by L. Mi and coworkers [91]. This CD sensor presenting carboxyl, hydroxyl, and amide groups on the surface was prepared by a solvothermal reaction from citric acid and urea. The different groups on the CD surface undergo changes with the pH environments leading to different fluorescence lifetime values in the pH range of 2.6–8.6 inside HeLa cells microenvironment [91].

Upconverting nanoparticles (UCNPs) are nanoscale particles exhibiting an absorption mechanism of two or more incident low energy photons and their conversion into a single emitted photon with higher energy. This mechanism is known as upconversion [60]. As consequence of this behaviour, an absorption of two photons in the infrared region will result into an emission in the visible spectrum region. UCNPs can function as pH VS for imaging purpose and can be designed by coupling a chemically sensitive dye and a quencher [60]. A rare example of UCNP pH VS was developed by E.A.H. Hall and coworkers and it is composed of two anthraquinone dyes, calcium red (UCNP-CaR) and alizarin red S (UCNP-ARS), mixed into a UCNP core with different ratios [92]. As a result, adducts with a different core shell dimensions are obtained [92]. When an UCNP-anthraquinone specific thick-shell cores was selected the pH sensor could be excited by NIR light and showed suitability for pH measurement in the range 4.0–6.4 [92]. This sensor could potentially enable for deep biological tissue and reduced background fluorescence.

An interesting class of pH VSs is represented by stimuli responsive nanoparticles known as hydrogels, obtained by organic molecules able to form supramolecular aggregates [93,94]. Among recently developed hydrogels, a pH VS, developed by X. Cao and coworkers, is able to change color and/or emission pattern [95]. This hydrogel is formed by 3-hydroxy-1,8-naphthalimide-based molecules branched with alkylamines of different length. The hydrogel system obtained from several organic solvents exhibited ICT bands and emission changes as function of the pH. Shorter alkylamine branches result in hydrogels tested for intracellular pH variation. When excited with a radiation wavelength of 350 nm, the most active hydrogel shows two emission peaks at 398 and 572 nm, respectively. A good linear correlation over the pH range 3.0–11.0 was obtained by evaluation of the fluorescence emission intensity ratio at the two emission wavelengths. A recent example of a biocompatible hydrogel from bacterial cellulose (BC), a carbohydrate-based material synthesized by bacteria, and incorporated of carboxymethyl cellulose (CMC), a negatively charged biocompatible polyelectrolyte, was developed by V.P. Hoven and coworkers [96]. The BC/CMC hydrogel was incorporated within a universal pH indicator and applied as colorimetric pH. The BC/CMC-based pH sensor proved to be useful for pH 4.0–9.0 in biological fluids (such as sweat). The sensor exhibits a fast colorimetric response easily detected by naked eye gradually turning from orange (pH 4.0) to deep-blue (pH 9.0) including all the intermediate colors. Interestingly, the hydrogel is functions as a colorimetric sensor for enzymatic glucose.

## 5. Bioengineered Visual pH Sensors

Measuring of the intracellular pH is very important since as it plays a vital role for cells regulation [97]. Proton gradient are the basis of the function of subcellular locations organelles such as lysosomes and mitochondria [97,98]. Notably, pH variation represents the driving force used by synaptic vesicles for shift in membrane electric potential [99,100]. Variation of pH is coupled to the cell cycle and apoptosis processes [101]. Protein-based pH VSs are genetically encoded (GE) sensors resulting from a translation of DNA sequence. GE encoding genes contained in a plasmid DNA are incorporated into tissue cells through techniques of transfection, electroporation, or viral vector injection transduction. Genetically encoded pH VSs are designed for fluorescence imaging of living cells and allow for simultaneous in vivo intracellular and extracellular pH measurement. These proteins function as intracellular pH detectors must not be cytotoxic, nor invasive; and should not interfere with cellular metabolic pathways [102]. For medical purpose the ability to reveal the pH in microenvironment for diagnostic purpose of cancer and allow for selective efficiency for live-cell microscopy [103,104,105]. A variety of protein-based pH VSs were developed since the discovery of the amazing properties of the green fluorescent proteins (GFPs, see Section 5.1). Other bioengineered protein-based pH VSs were obtained as hybrid materials by mixing proteins with organic dyes, or by protein immobilization on suitable substrates, reviewed in Section 5.2.

### 5.1. Genetically Encoded Visual pH Sensors

Green fluorescent proteins (GFP) are able to convert blue/ultraviolet radiation absorbed from light to visible light (green fluorescence) [106]. For this amazing property GFPs gained a widespread use for multiple purposes including imaging of cells [106]. At the core of its function a GFP contains a chromophore group shielded deep within a protein shell shaped like a braided wire shield [107]. This chromophore group is the result of post-translational modification involving an intramolecular cyclization of three protein amino acids generally including a central tyrosine or a tryptophan amino acid (Table 1) [108]. Biochemical research with GFPs inspired re-engineering of these macromolecules with the aim to deliver stable and specifically sensing macromolecules giving-off luminous fluorescent colors besides green [109,110]. A great variety of engineered GFP mutants have been designed to obtain different and stable absorption/color emission [111,112]. Protein crystallography is instrumental in understanding the function of pH sensing proteins, protonation and deprotonation effect, and support engineering of new mutants such of GFPs for intracellular pH detection [97,113]. A number of studies from pioneering work of G. Miesenböck and coworkers was devoted to the engineering of pH-sensitive GFPs or other pH sensing protein [114]. These re-engineered proteins are often aimed to sense pH of cellular compartments far from physiological pH of ~ 7.4 (extracellular/physiological pH value), improve cellular penetration, and gain a better control over subcellular targets [114,115,116,117,118]. Recent developments for cells imaging using re-engineered GFP-like proteins insensitive to acidic environment of organelles for degradation of biomolecules was reviewed by T. Nagai and coworkers [119]. Protonation of the chromophore in the studied GFPs could cause decreasing of fluorescence quantum yield, or contraction of electron delocalization and/or stability, or shift of the absorption band towards UV spectral region [119]. To better understand optical properties of GFPs as function of the pH, a recent structural analysis revealed the importance of the chromophore orientation with respect direction of absorbed light and emitted by common [120]. A summary of three-dimensional structures of commonly used GFPs for pH sensing is given in Table 1. A modified tyrosine residue is often present as central chromophore’s residue, although tryptophan seems to be present in GFPs stable at low pH [121].

**Figure 3:**
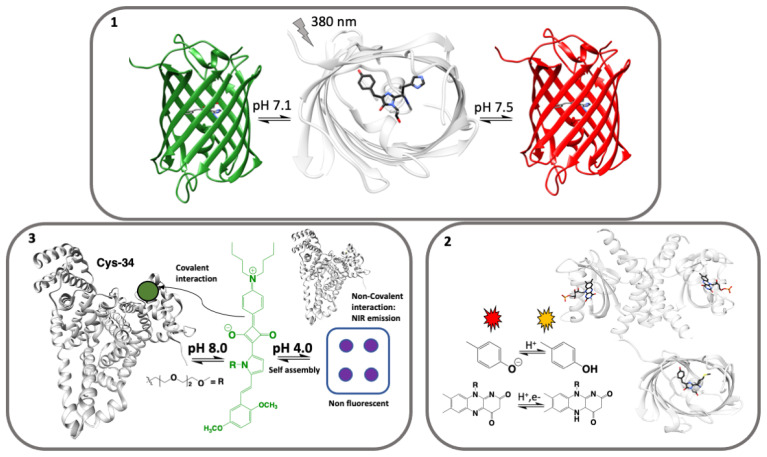
Bioengineered visual pH sensors. Box 1. Ribbon plot of Dendra2 protein [129]: chromophore appears as sticks (middle figure, nitrogen blue, oxygen red), accession code 2vzx [124]. Box 2. Ribbon plot representation of chimeric protein pHaROS [130] comprised of LOV protein, bound to its cofactor FMN (sticks), accession code 4hj3 [131], and GFP variant of *mkate*, accession code 3rwa [132]. A peptide linker is connecting LOV to *mkate* variant. Figure made with “UCSF Chimera” [133] for rendition purpose only and neither derived from experimental determination, nor theoretical calculation. Chemical structures of chromophore’s phenol group and flavin mononucleotide are represented. Box 3. Ribbon plot of bovine serum albumin, accession code 4f5s [134]. Under basic condition the sensor is linked to the side chain of Cys-34 and green fluorescence is produced [135,136]. Under acidic condition, the sensor can self-assemble (purple spheres) producing a non-fluorescent aggregate. Adapted from references indicated in square brackets.

Recent examples of GFPs used as pH sensors among those reviewed below are shown in Figure 3.

A natural fluorescent protein Dendra2 was photoconverted from green- to red-emitting protein as function of the pH by A.A. Pakhomov and coworkers. They used a radiation with a wavelength of 380 nm to promote Dendra2 to an excited state (see Figure 3, box 1) [129,137]. Upon photoconvertion, the protein color emission turns back to green in acidic medium (pH < 7.1), due to the protonation of chromophore phenolic group. At pH > 7.5 Dendra2 emits red color and was used as a ratiometric pH-sensor for HEK 293 and chinese hamster ovary (CHO) cells imaging within the physiological pH range.

Genetically-encoded proteins were also obtained by insertion of pH responsive fragments in suitable biological scaffold to match a local pKa protein microenvironment and fluorescence response [102,138]. This is particularly useful for imaging of synaptic transmission mediated by rapid fusion of synaptic vesicles with the plasma membrane with a concomitant pH variation.

A semisynthetic pH-sensitive protein-conjugate combining a GFP with a red-shifted difluorinated fluorescein derivative, so-called “Virginia Orange” (VO), was developed by D. Perrais and coworkers [12]. This bioconjugate sensor exhibits ideal properties for imaging detection and pH variation (from extracellular pH of 7.4) upon vesicle fusion and monitoring exocytosis in living cells. A similar approach was developed by J. Klingauf and co-workers to obtain a fluorescent conjugate used for optical detection of synaptic vesicle [139]. As an alternative to GFPs, a fluorescent organic dye as pH sensor, the commercially available phospholipidic organic dye known as cypHer5E, was covalently bound to lipid molecules through lipid-dye coupling and able to signal [139]. 

A GFP can also be used as a whole FRET block, used in conjunction to another GFP, to design a chimeric construct where the two GFPs are linked together. One of these two GFPs will influence or transfer the emission from one GFP to the other counterpart. Using this approach, R. Malli and coworkers engineered a pH VS consisting of a chimeric protein obtained by linking (through a flexible linker) mTurquoise2, a cyan fluorescent protein (FP) variant, to EYFP, a pH sensitive and enhanced yellow fluorescent protein [126,140,141]. The pH “reporter” named pH-Lemon is used as a ratiometric pH sensor optimized for live imaging of acidic cellular compartments. pH lemon functions through a FRET mechanism. Under neutral/basic conditions pH-Lemon undergoes a FRET enhancement of the emission in that mTurquoise2 undergoes to a FRET transfer to the EYFP which in turn emits a bright yellow emission (525 nm). On reverse, under acidic pH no FRET mechanism is observed. Therefore, upon excitation with 430 nm radiation, pH-Lemon EYFP undergoes a decrease of yellow fluorescence, while mTurquoise2 produces a cyan emission (475 nm). In summary, this sensor gives-off a yellow emission in neutral/basic condition and yellow emission in acidic pH. Interestingly, the sensor can detect neutral and acidic vesicles and cell compartments of different pH values throughout the endomembrane system, proving its usefulness for monitoring of local pH dynamics of subcellular microstructures in living cells. Most of FPs used for living cell pH determination are variants of green and yellow fluorescent proteins, not spectrally compatible for dual-compartment imaging. Using a double mutant (I158E/Q160A) of the red fluorescent protein mCherry (pdb entry ID 3nt3), M. Tantama and coworkers developed an effective ratiometric pH sensor with a pKa of 7.3 [128,142]. This variant of mCherry protein shows an activity and metabolism-dependent pH dynamics in cultured primary neurons and neuroblastoma cells. The pH VS was used to measure compartments specific pH changes in combination with GFP pH sensors. As of this writing, P. Xu and coworkers published a study of an engineered red FP (named pHmScarlet) with high pH sensitivity enabling imaging for vesicles docking and exocytosis [143]. pHmScarlet was developed to overcome limitations of Superecliptic pHluorin (SEP), a classical variant of green fluorescent protein largely used for studies of synaptic vesicles [144]. Dual-color imaging by combination of pHmScarlet and SEP was possible [143].

Inspired by protein-based genetically encoded pH VSs B. Borhan and coworkers performed an innovative study for re-engineering of protein binding of cellular retinoic acid in order to use a modified retinol (protein substrate) as a chromophore for radiation absorption [145]. The re-engineered protein named CRABPII was targeted for the binding of a protonated Schiff-base of retinal, that confers a colorimetric pH sensing to the protein, and enables a ratiometric mode sensing system. A genetically encoded dual-function sensor by using a pH sensitive chimeric protein comprised of a variant of “light-oxygen-voltage” (LOV) sensing protein and a variant of GFP red fluorescent protein (Figure 3, box 2) was developed by C.-P. Song and coworkers [130]. The protein LOV forms a dimer and each subunit contains a non-covalently bound flavin mononucleotide (FMN) with the function of shuffling electrons for signal transduction [131]. The chimera protein named pHaROS (pH-and redox-sensitive fluorescent protein, or iLOV-mBeRFP) enables a cross simultaneous real-time detection of a redox-potential and pH detection in living cells. The protein pHaROS consists of two proteins linked together: a plant phototropin2 iLOV, an enhanced mutated LOV (C426A) fluorescent protein, covalently linked (through a peptide linker) to a blue light-excited red fluorescent protein, mBeRFP (known as “mkate” variant) (Figure 3, box 2) [125,131]. Images of yeast strain INVSc1cells treated with buffers at pH 5.0–9.0 reveals signal emission with wavelengths of 590 to 630 nm [131].

### 5.2. Bioengineered Materials Visual pH Sensors

Recent preparation of pH VSs consists of hybrid materials including nanoparticles mixed to proteins [146]. A protein-organic dye mixture was developed by A. Ajayaghosh and coworkers [135,136]. The dye squaraine was selected to study its fluorescent properties in presence of human or bovine serum albumin, a carrier protein circulating in the blood plasma and widely used for research purpose (Figure 3, box 3). Squaraine can self-assemble through non-covalent interactions at pH 4.0. Under this condition, the mixture shows a NIR emission, but not fluorescent signal while interacting with protein. On the other hand, squaraine can disassembly at pH 8.0 and bind covalently to bovine serum albumin (through residue Cys-34), resulting in a fluorescent adduct emitting at 480 and 700 nm, respectively. Even more, a specifically sensed pH window can be selected by choosing a different serum albumin-squaraine stoichiometric ratio. Among these ratios the adduct composition designed for an optimal signal was used for fluorescence imaging of HeLa cells and to monitor pH variation.

As discussed in Section 4.2 immobilization of a sensor onto the porous surface of solid matrices is an approach to produce a pH-VS. Similarly, a fluorescent protein within a heterogeneous material was studied by B. Nidetzky and coworkers [147]. They developed a nanosized pH VS by homogenously immobilizing a GFP onto the surface of porous materials or metal chelates [147]. An enhanced fluorescent and stable yellow fluorescent protein (abbreviated as sYFP) was immobilized onto supports of Ni-Ag metal chelates or Ag-Gly (glycine on silver-nanoparticles). In addition, traditional materials such as agarose, silica (glass) and methacrylate were employed for protein immobilization. These protein nano composites result in ratiometric sensor that could potentially be used for sensing in biological systems in response to pH variation between 6.0 and 8.4.

## 6. Conclusions

Measuring of pH is a fundamental task for a variety of research areas ranging from chemical to environmental and biomedical sciences. We reviewed a specific class of optical sensors able to change their color and/or fluorescence emission. The common definition of “visual sensor” refers to the eyes as visualization tool. pH VSs are chemical and biochemical probes resulting in a signal detected by eyes that upon analysis allows for pH measurement. Real-time analysis, non-invasiveness, low-cost methods are unquestionable advantages of pH VSs over conventional spectrophotometric and electroanalytical analysis.

With this review we provide an overview of pH VSs as colorimetric, fluorescent, or dual-mode responsive sensors. These sensors include molecular synthetic organic sensors, those based on metal organic framework (MOF), engineered sensing nanomaterials, and bioengineered sensors. We review different typological chemical entities of pH VSs, their three-dimensional structures, signaling mechanisms for pH sensing and applications from a chemical point-of-view up to in vivo biochemical applications. Bioengineered pH VSs able to monitor pH of specific cells or subcellular localization are useful for understanding aspects of cellular functions and for medical diagnostic. The progression of this review from simple organic molecules to biological macromolecules seeks to benefit beginners and scientists embarking on a project of pH sensing development, who needs background information and a quick update on advances in the field. Lessons learned from these tools will aid pH determination projects and provide new ways of thinking for cell bioimaging or other cutting-edge in vivo applications.

## Figures and Tables

**Table 1:** New generation of commonly used GFPs for pH sensing. The entries are retrieved from the Protein Data Bank [122]. Identifiers of chromophore and harboring three-amino acids are indicated. The 3D structure for each entry and other protein annotations can be explored from the following link and searching for the indicated PDB code. Related entries can be retrieved from the structure summary page of each entry. Link: https://www.rcsb.org, accessed on 31 March 2021.

Green Fluorescent Protein Name	PDB Entry Code and Chromophore Cif Code Identifier	Amino Acids Undergoing Post-Transitional Modification and Resulting in a Specific Chromophore
Ratiometric pHluorin	5j3n: GYS [123]	GYS: SER, TYR, GLY
Dendra2	2vzx: CR8 [124]	CR8: HIS, TYR, GLY
mKate (variant)	3bxa: NRQ [125]	NRQ: MET, TYR, GLY
Cyan FP SCFP3A (K206A)	2ydz: CRF [126]	CRF: THR, TRP, GLY
Cyan FP SCFP3A	5ox9, 5ox8: B2H [117]	B2H: THR, TRP, GLY
SHardonnay	3v3d: CR2 [127]	CR2: GLY, TYR, GLY
mEos4b	6yls: 5SQ [120]	5SQ: HIS, TYR, GLY
mCherryEA	3nt3, 3nt9: NRQ [128]	NRQ: MET, TYR, GLY
mRuby	3u0l, 3u0m: NRQ [118]	NRQ: MET, TYR, GLY
mTurquoise2 (K206A)	3ztf: SWG [126]	SWG: SER, TRP, GLY

## Data Availability

Data are available in this review, including link to database and code identifiers for three-dimensional structures.
